# Multiple imputation methods for longitudinal blood pressure measurements from the Framingham Heart Study

**DOI:** 10.1186/1471-2156-4-S1-S43

**Published:** 2003-12-31

**Authors:** Terri Kang, Peter Kraft, W James Gauderman, Duncan Thomas

**Affiliations:** 1 Department of Preventive Medicine, University of Southern California, Los Angeles, California, USA; 2 Department of Biostatistics, University of California at Los Angeles, Los Angeles, California, USA

## Abstract

Missing data are a great concern in longitudinal studies, because few subjects will have complete data and missingness could be an indicator of an adverse outcome. Analyses that exclude potentially informative observations due to missing data can be inefficient or biased. To assess the extent of these problems in the context of genetic analyses, we compared case-wise deletion to two multiple imputation methods available in the popular SAS package, the propensity score and regression methods. For both the real and simulated data sets, the propensity score and regression methods produced results similar to case-wise deletion. However, for the simulated data, the estimates of heritability for case-wise deletion and the two multiple imputation methods were much lower than for the complete data. This suggests that if missingness patterns are correlated within families, then imputation methods that do not allow this correlation can yield biased results.

## Background

Genetic analyses can be affected by missing data in two ways. First, there can be a loss of efficiency due to reduced sample size if potentially informative subjects are completely excluded from the analysis because one or more variables are missing in the model. Second, and more serious, results can be biased if missingness itself is related, directly or indirectly, to some of the relevant factors. These problems are particularly germane to longitudinal studies, because few subjects will have complete data for all visits, and the fact that missingness could be an indicator of an adverse outcome (e.g., premature death due to heart disease or study drop-outs related to failure to comply with hypertension treatment). In a genetic analysis, it is also possible that missingness patterns could be correlated within families, which leads to distortion of estimates of familial aggregation.

The problem of missing data has received considerable attention in the statistical literature (for reviews, see [1-3]), particularly in the context of longitudinal data [4], but has seldom been applied in genetic analyses. One method to deal with missing data in the analysis is multiple imputation, in which several augmented data sets are generated by random replacement of missing values with samples from appropriate distributions in order to obtain more stable estimates of the parameters of interest and to quantify the contribution to the variance of the parameter estimates from the uncertainty in the imputation.

Data are said to be missing at random (MAR) if the probability of missingness is independent of all unobserved data, although it *can *depend upon observed data. In the context of longitudinal studies, we further distinguish two subtypes: "intermittent" missingness, in which some observations of a repeated measure are missing followed by later observations, and "premature truncation", in which once an observation is missing all subsequent observations are also missing. The latter missing type is likely to be more serious because the reason for missingness could be relate to vital status or other important determinants of the outcome. Hence, estimates of long-term levels or rates of change could be biased if unusual values tend to be systematically missing. Data with no intermittent missingness are said to have a "monotone missing data pattern".

In a real life situation, it is not possible to verify whether data are MAR since we cannot compare the observed and unobserved values-we can only hope that it is the case with the data at hand. Classical methods for multiple imputation available in off-the-shelf statistical packages such as SAS assume "ignorable missingness". In other words, data are MAR and the parameters that describe the probability of missingness conditional on the observed data are distinct from inferential parameters (see Little and Rubin [1], pp. 117-120). There are more modern approaches that do not assume "ignorable missingness". However, these require a model for the joint distribution of the data and the specification of the missingness process (see Little and Rubin [1], pp. 327-331). Also, these modern approaches often require sophisticated statistical procedures such as Gibbs sampling for implementation, and results will be sensitive to the choice of the missingness model. As Allison [5] notes, "I won't say don't go there, but if you do, proceed with caution."

In this paper we compare and discuss two multiple imputation methods that can be done using the standard SAS software [6].

## Methods

### Subjects and phenotypes

We analyzed both real and simulated (replicate 59) data sets. Each data set comprised two cohorts: the "original cohort," enrolled in 1948, was examined every two years for total of 21 visits; the "offspring cohort," enrolled in 1971, was examined every four years (following an initial 8-year interval) for total of 5 visits. We analyzed the phenotype systolic blood pressure (SBP), together with covariates cohort, age at exam, sex, hypertension treatment (HRX), and body mass index (BMI). All subjects with phenotype data on at least three visits (*N *= 2583 and 2686 from two cohorts combined for the simulated, S, and real data, R, respectively) were included in this analysis. The average number of intermittent missing SBP observations was 0.88 (SD = 1.97, ranging from 0 to 17) in Cohort 1 for the simulated data, and 0.68 (SD = 1.70, ranging from 0 to 12) for the real data. For Cohort 2, the means were about 0.20 and 0.06 (both SD = 0.7, ranging from 0 to 2). For the last visits, only 41.3% (R) and 21.5% (S) had SBP values in Cohort 1, whereas most of Cohort 2 returned (both 90.9%).

Since exams were scheduled at regular intervals and height and weight did not vary much from visit to visit, we imputed intermittent missing values in these variables using deterministic rules (missing data due to premature truncation were not imputed). For Cohort 1, missing ages were imputed by adding two years to the age at the previous visit; for Cohort 2, four years were added to the age at previous visit, except for the second visit, when eight years were added to the age of the first visit. If the imputed value was greater than or equal to age at the next visit, three years were subtracted from the age at the following visit. Missing heights and weights were replaced by the most recent value. Several subjects had no height information (three in the real data and one in the simulated data), so we estimated their heights by single imputation, based on a regression of height on weight from subjects with available data.

### Multiple imputation

We assumed that SBP values were MAR and multivariate normal. Multiple imputation inference assumes that the model used to analyze the imputed data (the analysis model) is the same as the model used to impute missing values (the imputation model) [7]. However, this is not a critical assumption as long as the variables that appear in one model but not in the other are not related to the dependent variable, and additional variables can be used to improve the imputation that are not needed in the analysis model [5]. In our analyses, age, BMI, HRX, and SBP were included in the imputation, but only sex, BMI, and SBP were included in the analysis model. Hypertension treatment was addressed separately before the analysis, as described below.

Two different imputation methods, propensity score [8,9] and regression methods [3,10], were compared to address the problem of potentially informative missingness. We also compared the two imputation methods to the case-wise deletion method for real data and complete data analysis for simulated data. The propensity score and regression methods require a monotone missing data pattern. Since these data had an arbitrary missingness pattern (for example, HRX was missing when SBP was observed and/or SBP at visit 4 was missing when SBP at visit 5 was observed), we applied these methods in a "time-wise" two-stage manner to data sets with intermittent missing values. First, we dealt with missing HRX and SBP at each visit chronologically. HRX was imputed first using age and BMI at that visit *t *and SBP from all previous visits *t*^- ^since HRX at visit *t *only depends on HRX and SBP at visit *t*^-^. Then SBP at visit *t *was imputed, conditional on recorded or imputed HRX at visit *t*. Only the intermittent missing data (subjects who returned for subsequent visit) were imputed; further "premature truncations" were not imputed.

The propensity score method is a semiparametric approach, based on the following steps. First, for each variable with missing values, a logistic model is fitted for the probability of missingness (the "propensity score") as a function of all previous variables in the data set. The observations are then grouped based on these propensity scores, and an approximate Bayesian bootstrap imputation is applied to each group. (This is done first by drawing a sample with replacement from the set of nonmissing observations, and then assigning the missing observations by sampling from this subset of nonmissing values.)

The regression method is a parametric approach, in which a regression model is fitted for each variable with missing values, using the previous observations as covariates. Based on the fitted regression coefficients, a new regression model is simulated from the posterior predictive distribution of the parameters and is used to impute the missing values for each variable. The process is repeated sequentially for variables with missing values. The regression method yielded a continuous value for imputed HRX, which was then converted to a binary variable as follows: the imputed value less than or equal to 0 was assigned as 0, and the value greater than or equal to 1 was assigned as 1 in the final imputed value. If the value was in between 0 and 1, the subject was assigned to treatment with corresponding probability. For those subjects who were either known or imputed to have received hypertension treatment at a given observation, SBP was adjusted further with Levy's algorithm to estimate their untreated SBP [11].

For comparison purposes, we also used the imputed and adjusted SBP values to form age-interval-specific residuals as in the GAW13 contribution from Kraft et al. [12]. We first averaged each subject's imputed SBP and BMI measurements over the age interval 35-50. Then the average imputed SBP was regressed on gender and average BMI. We used the residuals from this regression in a variance-components analysis with a fixed mean effect, a random additive polygenic effect, and an independent random error. Thus there were three parameters to estimate: the mean μ, the polygenic variance σ_a_^2^, and the random error variance σ_e_^2^.

Ten imputed data sets were generated for each of the three methods for the real and simulated missing data, and each was analyzed using the variance components method. The results from the multiple analyses were then combined for final summary. Parameter estimates are given by simple averages of the estimates over all imputations. The within-imputation variance  (the mean of the sampling variance estimates from each imputation) and the between-imputation variance *B *(the sample variance of the estimates across imputed data sets) were calculated, and the total variance is given by



where *m *is the number of imputations. The relative increase in variance due to nonresponse [3] is calculated by



## Results

From the simulated data, the estimates of the polygenic and random environmental effects from the complete data were strikingly different than the estimates from case-wise deletion and imputations. The estimated heritability from the complete data was 81%; case-wise deletion, the propensity score, and regression methods yielded much smaller heritabilities (0.13, 0.11, and 0.12, respectively; Tables 1 and 2). The standard errors from both multiple-imputation analyses were smaller than from the case-wise deletion analysis.

For the real data, both multiple imputation methods yielded similar conclusions to case-wise deletion (Table 3). Estimates of the polygenic effect were significantly different than zero, with an estimated heritability of 0.34 for both imputed and case-wise deleted data. The within-imputation variance for the parameter estimates was smaller than the case-wise deletion variance (the square of the standard error from Table 3), reflecting the fact that the imputed observations contribute information to each analysis (Table 4). However, due to the added imputation sampling error *B*, standard errors of parameter estimates from the imputation methods need not be smaller than the standard errors from case-wise deletion. In this case the standard errors from the propensity-score method were slightly larger.

## Discussion

Case-wise deletion and multiple imputation analyses of the simulated data severely underestimated the polygenic effect and overestimated the random environmental effect relative to the complete data. This may be due to strong correlation in missingness within families, as described in Daw et al. [13], as well as non-ignorable missingness induced by premature truncation. Since we imputed data independently for each subject, we were not able to allow for this dependency. Imputation methods that take familial relationships into account may perform better; such methods were discussed in the GAW13 contribution by Fridley et al. [14]. On the simulated data, their estimates were much more consistent between imputed and complete data than our estimates. Their complete data estimates were also noticeably different than ours (their estimated heritability is about 35% – less than half of ours), perhaps because they used cross-sectional data (whereas we used age-matched data) and our SBP trait is an average of several visits, which should reduce measurement error.

We did not observe clear differences in performance between multiple imputation and case-wise deletion in these applications. In part, for example, this is due to the fact that only 10 subjects in the real data failed to contribute any data in this age range due to intermittent missingness, so the multiple imputation methods use only 0.4% more subjects (2409 vs. 2419).

This application of multiple imputation methods to real data cannot of course address the statistical performance (bias, power, etc.) of the approach. Unfortunately, the complexity of the simulation model, with many intermediate traits and time-dependent effects, precludes determining a "true" heritability for comparison with our estimated values. However, much is known about the performance of multiple imputation method in other contexts. Under MAR, parameter estimates produced by multiple imputations have been shown to be consistent, asymptotically efficient, and asymptotically normal [7]. The propensity score approach is attractive because it is semiparametric, but both methods require the MAR assumption. Our results from the simulated data suggest that results are very sensitive to this assumption. If there is a reason to believe the MAR assumption does not hold, alternative methods should be used. One such approach, discussed during the GAW13 but not implemented by any of the participants, is marginal structural models [15] (a form of weighted regression using ratios of estimated probabilities of missingness as weights), which appears to merit further attention. However, this method requires data to be missing completely at random (a more stringent condition than MAR in which the probability of missingness cannot depend on observed data) within each weighting class. Computation of appropriate standard errors is also less straightforward and no statistical package is available for more complex situations [1]. Similar techniques could also be used to adjust time-dependent covariates as well as intermediate variables, like hypertension treatment [16].

Imputation techniques for non-ignorable missingness are also available, but require stronger modeling assumptions regarding the nature of the missingness mechanism [7]. Clearly, methods that can allow for both longitudinal and familial dependencies simultaneously would be desirable.

## Figures and Tables

**Table 1:** Estimates of variance components (simulated data)

		Estimates	
		
	Polygenic Heritability Score	Polygenic Variance (SE)	Random Error (SE)
Complete data	0.81	126.8 (7.51)	30.3 (4.01)
Case-wise deletion	0.13	21.6 (5.93)	146.4 (6.91)
Propensity score	0.11	18.8 (5.75)	145.5 (6.81)
Regression	0.12	20.4 (5.85)	147.5 (6.88)

**Table 2:** Variance information on multiple imputations (simulated data)

	Between	Within	Total	Relative Increase in Variance
Propensity score				
Polygenic component	0.89	32.02	33.01	0.031
Random error component	1.15	45.10	46.37	0.028
Regression				
Polygenic component	0.33	33.89	34.25	0.011
Random error component	0.42	46.87	47.33	0.010

**Table 3:** Estimates of variance components (real data)

		Estimates	
		
	Polygenic Heritability Score	Polygenic Variance (SE)	Random Error (SE)
Case-wise deletion	0.34	76.1 (9.24)	149.3 (8.37)
Propensity score	0.34	75.2 (9.27)	148.9 (8.52)
Regression	0.34	75.1 (9.16)	148.3 (8.36)

**Table 4:** Variance information on multiple imputations (real data)

	Between	Within	Total	Relative Increase in Variance
Propensity score				
Polygenic component	2.34	83.34	85.91	0.031
Random error component	3.66	68.63	72.66	0.059
Regression				
Polygenic component	0.69	83.22	83.98	0.009
Random error component	1.42	68.39	69.95	0.023
